# Isolation of multipotent progenitor cells from pleura and pericardium for tracheal tissue engineering purposes

**DOI:** 10.1111/jcmm.16916

**Published:** 2021-11-01

**Authors:** Rayna de Wit, Sailay Siddiqi, Dorien Tiemessen, Rebecca Snabel, Gert Jan Veenstra, Egbert Oosterwijk, Ad Verhagen

**Affiliations:** ^1^ Department of Cardio‐thoracic surgery Radboud University Medical Center Nijmegen the Netherlands; ^2^ Department of Urology Radboud Institute for Molecular Life Science Radboud University Medical Center Nijmegen the Netherlands; ^3^ Department of Molecular Developmental Biology Radboud Institute for Molecular Life Science Faculty of Science Radboud University Nijmegen the Netherlands

**Keywords:** airway reconstruction, stem cells, tissue engineering, tracheal replacement

## Abstract

Tissue engineering (TE) of long tracheal segments is conceptually appealing for patients with inoperable tracheal pathology. In tracheal TE, stem cells isolated from bone marrow or adipose tissue have been employed, but the ideal cell source has yet to be determined. When considering the origin of stem cells, cells isolated from a source embryonically related to the trachea may be more similar. In this study, we investigated the feasibility of isolating progenitor cells from pleura and pericard as an alternative cells source for tracheal tissue engineering. Porcine progenitor cells were isolated from pleura, pericard, trachea and adipose tissue and expanded in culture. Isolated cells were characterized by PCR, RNA sequencing, differentiation assays and cell survival assays and were compared to trachea and adipose‐derived progenitor cells. Progenitor‐like cells were successfully isolated and expanded from pericard and pleura as indicated by gene expression and functional analyses. Gene expression analysis and RNA sequencing showed a stem cell signature indicating multipotency, albeit that subtle differences between different cell sources were visible. Functional analysis revealed that these cells were able to differentiate towards chondrogenic, osteogenic and adipogenic lineages. Isolation of progenitor cells from pericard and pleura with stem cell features is feasible. Although functional differences with adipose‐derived stem cells were limited, based on their gene expression, pericard‐ and pleura‐derived stem cells may represent a superior autologous cell source for cell seeding in tracheal tissue engineering.

## 1 INTRODUCTION

Airway obstruction can be due to a variety of pathologic conditions ranging from traumatic injury to inflammatory diseases and tumour formation (both benign and malignant) resulting in respiratory distress. Current treatment for these patients is surgical resection of the affected segment followed by end‐to‐end anastomosis.^1^, ^2^, ^3^ However, tracheal involvement of more than 30% in children and 50% in adults is considered to be inoperable due to the limited length available, resulting in unacceptable anastomotic tension.^4^, ^5^ For these patients, alternative treatment options are lacking. Artificial substitutes often lack the appropriate biological composition to support cell growth.^6^, ^7^ A recent development is the use of aortic allografts, but long‐term follow‐up and validation is needed to show their value in clinical practice and find out whether complications such as calcification and degeneration will occur.^8^, ^9^ Allogenic transplantation of tracheas is limited by the lack of donors, as well as the need for immunosuppressants, undesirable in oncological patients.^10^, ^11^ Although matrices of decellularized tracheas do support cell adherence and ingrowth,^12^, ^13^ these constructs fail long term due to inadequate mechanical support and insufficient revascularization. Moreover, immunological responses caused by incomplete removal of cell remnants may occur.^14^, ^15^ Attempts to create a long‐term solution using tissue engineering (TE) techniques have not been successful so far.

Failure to successfully create a functional trachea may be due to both cell choice and matrix choice. For the development of a sustainable construct, the right cell source and cell type, proper differentiation stimuli and a suitable scaffold are essential.^16^, ^17^, ^18^ Thus far, the ideal source for cartilage progenitor cells is still unknown.

In tissue engineering, the cellular origin subdivision is based on the embryonic layers: ectoderm, endoderm and mesoderm.^19^ This subdivision was generated as it is pivotal to fundamentally understand tissue origin prior to attempts to imitate the natural process of tissue development.^20^, ^21^ Mesenchymal stem cells, especially derived from bone marrow and adipose tissue, are often used in tissue engineering.^22^, ^23^ However, though these cells share a mesodermal origin, they are not highly related to those of the trachea.

The respiratory epithelium arises from the endodermal part of the respiratory diverticulum, while tracheal cartilage and smooth muscle cells, essential for a sustainable construct, grow from the lateral mesodermal layer. Since pericardium and pleura also arise from this layer, progenitor cells from these tissues may provide a better source for tracheal tissue engineering.

In the present study, we investigated the presence and relative value for tracheal tissue engineering of progenitor cells isolated from different tissues that originate from mesoderm, like the native trachea. Progenitor cells from these tissues may be used as a new and possibly superior cell source in tracheal tissue engineering.

## 2 MATERIALS AND METHODS

### 2.1 Animal information and related guidelines

Tissues were harvested from six landrace pigs (±50kg) according to the institutional guidelines of Laboratory Animal Research. This study was approved by the Ethical Committee on Animal Research of the RadboudUMC, the Netherlands. Tissues were harvested from animals with planned termination for non‐related studies.

### 2.2 Isolation and culture of progenitor cells

Immediately after sacrifice, trachea, pericard, pleura and adipose tissue were harvested and placed in sterile phosphate buffered saline (PBS) with 1% penicillin/streptomycin (P/S). Tracheal explants were placed in culture flasks. Pericard and pleura were digested with collagenase type I for 30 min at 37°C, and adipose tissue was digested with collagenase type II for 60 min at 37°C. Suspensions were washed with PBS, and cells were collected by centrifugation. Isolation was performed using plastic adherence within the culture flasks. Cells were cultured in complete αMEM culture medium (Gibco). Cell culture medium was changed every 3 days. When cultures reached 80% confluence, cells were harvested by trypsin digestion and seeded 1:3. Cells in passage 3 were harvested for further analysis.

### 2.3 RNA isolation and RT‐qPCR

Isolation of total RNA and synthesis of cDNA was performed according to manufacturer's manual (Invitrogen). Gene expression was evaluated using SYBR Green qPCR analysis with the LightCycler LC480 (Roche). HPRT and GAPDH mRNA expression was used for normalization. Relative gene expression of several stem cell‐related genes such as CD73, CD90, CD115, CD117 and SOX9 was calculated using the ΔΔCt‐method and compared to the control group of isolated adipose‐derived stem cells (ADSCs).^24^ Primer sequences are listed in Table S1.

RNA used for RNA sequencing was purified using RNA Clean and Concentrator column (Zymo), clean‐up, and purified RNA was run on a tricine/triethanolamine electrophoresis gel to assess the integrity.

#### 2.3.1 RNA‐sequencing library preparation and data analysis

Libraries were generated from 250ng RNA starting material using the KAPA‐RNA‐HyperPrepkit with RiboErase (Roche) to remove ribosomal RNA, according to manufacturer's instructions. Library amplification was performed with nine cycles, after which the size distribution was determined around 350 bp with Bioanalyzer (Agilent Technologies).

Paired‐end library sequencing was performed with NextSeq500 Illumina platform, at a 43 bp read length. Salmon v0.12.0^25^ quant was used to align reads to the Sus scrofa genome assembly 11.1 from Ensembl. R‐package DESeq2 v1.22.2^26^ was used to transform the transcript per million (TPM) abundancies to regularized log and determine the differentially expressed genes between the tissues (Wald test), by performing pairwise comparisons between all combinations. The differential gene list was filtered for a log2‐fold change >1 and *p*‐adjusted value <0.05, filtered for genes annotated in the Sus scrofa genome (a list of 520 significant differential genes) and visualized with pheatmap v1.0.12.

The R package clusterProfiler (v3.10.1) was used to perform Gene Ontology enrichment on the gene clusters.

### 2.4 Differentiation assays

Pluripotency was investigated by RT‐PCR and histological evaluation. Cells were chemically induced to differentiate and compared to a control group of cells cultured in standard αMEM.

Chondrogenic differentiation was induced using supplemented culture medium (DMEM‐HG, 10%FCS, 1%P/S, 1%ITS, 100 µg/ml Sodiumpyruvate, 40 µg/ml L‐proline, 50 µg/ml L‐Ascorbic acid, 1mM dexamethasone, 10ng/ml TGF‐B). Osteogenic induction medium consisted of αMEM, 10% FCS, 1%P/S, 0.1µmol/L dexamethasone, 0.05mmol/L‐Ascorbic acid and 2.4gr/L β‐glycerophosphate.

Adipogenic differentiation was induced using αMEM, 10%FCS, 1%P/S, 1 µmol/L dexamethasone, 0.5 mM 3‐isobutyl‐1‐methyl‐xanthine, 2 ml/L 100 mM indomethacin and 10 µmol/L recombinant human insulin. Cells were cultured for 14 days, and medium was changed every 3 days. Thereafter, cells were harvested and phenotypically analysed. Primer sequences are listed in Table S2.

#### 2.4.1 Histological evaluation of differentiation

Deposition of glycosaminoglycans (GAGs) after chondrogenic induction was evaluated using Alcian‐Blue staining (Sigma‐Aldrich). Osteogenic induction was assessed using Alizarin‐Red (Sigma‐Aldrich) staining to objectify the presence of calcium deposits. Oil‐Red‐O (Sigma‐Aldrich) staining was used to detect lipid deposits after adipogenic induction. All staining's were performed according to the manufacturer's manual.

### 2.5 Scaffolds

Collagen matrices (type I collagen) were prepared from bovine achilleas tendon (Southern Lights Biomaterials) as previously described.^27^, ^28^ In brief, a 0,5% (w/v) collagen suspension was made by swelling and subsequently homogenization in 0.25 M acetic acid at 4°C. The suspension was deaerated by centrifugation (at 120 g for 30 min), casted in 6‐well plates and frozen at −20°C. The scaffolds were lyophilized and subsequently chemically crosslinked for 4 hr with 33 mM 1‐ethyl‐3‐dimethyl‐aminopropyl carbodiimide (EDC) and 6 mM N‐hydroxysuccinimide (NHS) in 50 mM 2‐morpholinoethane sulphonic acid (pH 5.0) in the presence of 40% ethanol. The matrices were washed consecutively in 0.1 M Na2HPO4, 1 M NaCl, 2 M NaCl and demineralized water, disinfected and stored at −20°C.

#### 2.5.1 Cell seeding and survival assays

Because cells should adhere and proliferate on a template, isolated progenitor cells were seeded on scaffolds at a density of approximately 1 × 10^6^cells/cm^2^ for 24 h at 37°C in a polyHEMA (SantaCruz Biotechnologies) coated 6‐well plate to prevent adherence of cells to the wells. Seeded scaffolds were cultured for 7 days. Samples were snap frozen in TissueTek (Firma), sectioned and stained with haematoxylin and eosin (H&E) according to manufacturer's manual. Cell viability was assed using the WST‐1 assay (Sigma‐Aldrich) according to manufacturer's manual. Absorbance was measured at OD 450 nm using a microplate reader (Wallac 1420 Victor).

### 2.6 Statistical analysis

Statistical analysis was performed using GraphPad Prism software. Expressed values are all shown as mean ± SD. Minimum requirement for individual experiments was N = 3. To compare different groups, analysis of variance ANOVA with Bonferroni post hoc test was used, with a significance threshold of *p *< 0.05.

## 3 RESULTS

### 3.1 Characterization of isolated progenitor cells

**FIGURE 1:**
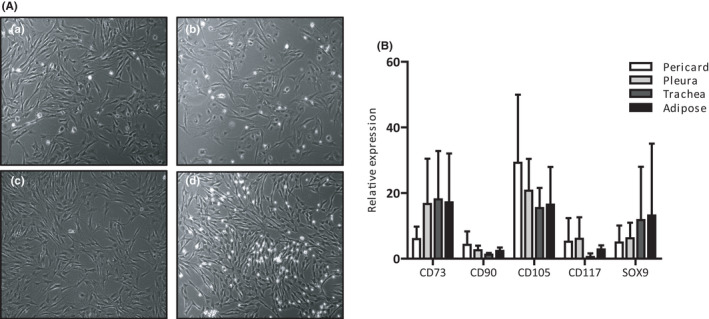
(A) Light microscopy of porcine progenitor cells at second passage isolated from (a) pericard, (b) pleura, (c) adipose tissue and (d) trachea. Original magnification 100×. (B) Characterization of cells from porcine pericard, pleura, trachea and adipose tissue. Relative expression of mesenchymal stem cell markers CD73, CD90, CD105, CKIT and SOX9 using RT‐PCR. No significant differences between groups were observed (*p *< 0.05) N = 6.

Isolation of cells from porcine pericard, pleura, adipose tissue and trachea was achieved using established protocols. In culture, cells formed a heterogeneous monolayer of adherent, spindle‐shaped cells (Figure 1A). After expansion, cells maintained this morphology for over five passages.

RT‐PCR analysis of the isolated cell populations for mesenchymal stem cell‐related markers showed expression of CD73 and CD90 and to a lesser extend of CD105 and CD117 in all isolated cell populations. SOX9, associated with cartilage formation and tracheal patterning, was also expressed (Figure 1B). Although differences in the relative expression of mesenchymal stem cell markers were observed, these were statistically insignificant.

### 3.2 Gene expression

**FIGURE 2:**
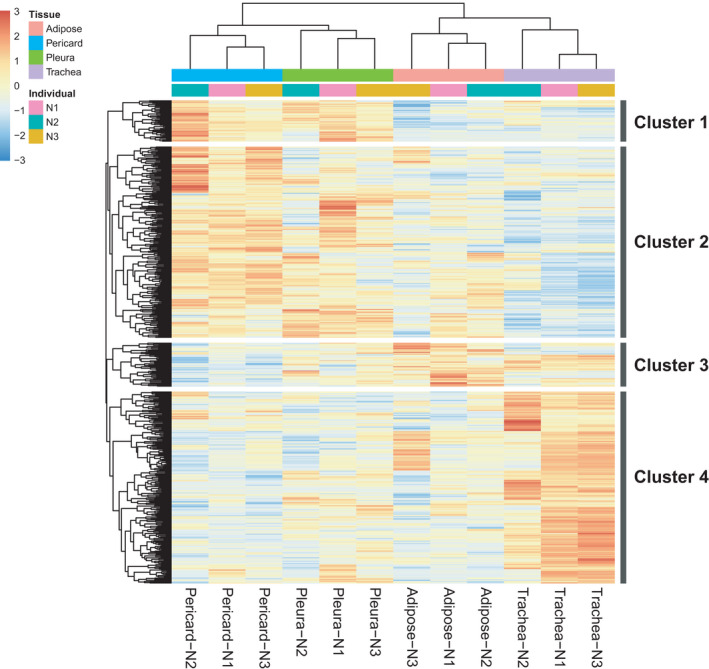
Heat map of RNA‐sequencing analysis for differentially expressed genes. Total RNA was extracted from isolated progenitor populations of pericard, pleura, adipose and tracheal cells, sequenced and analysed as described previously. The differential gene list was filtered for a log2‐fold change >1 and a p‐adjusted <0.05. N = 3.

**FIGURE 3:**
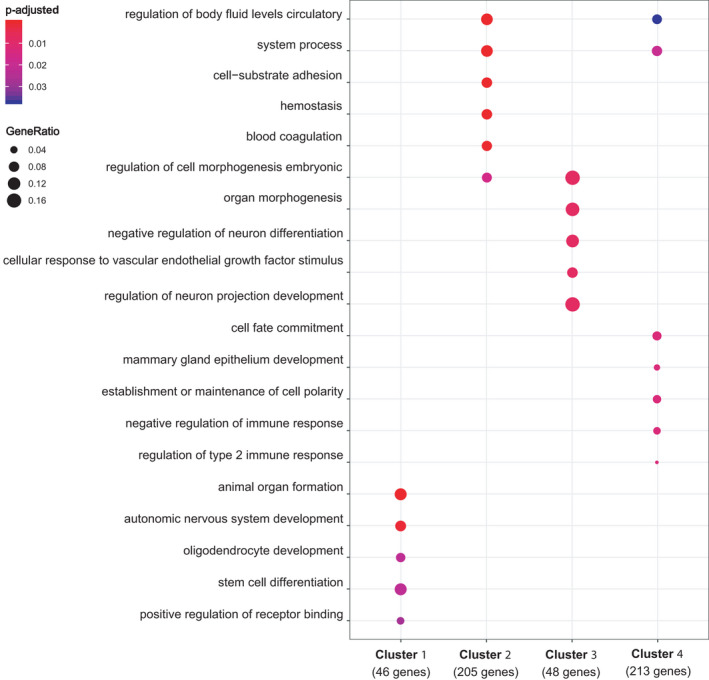
Gene ontology term analysis. Gene Ontology enrichment was performed on the gene clusters from isolated progenitor population of pericard, pleura, adipose and tracheal cells, sequenced and analysed as described previously. N = 3.

To test whether the expression profiles of pleura and pericard‐derived cells differed, possibly indicating commitment towards a particular differentiation pathway, we conducted RNA‐seq analysis of the isolated cell populations. Hierarchical clustering demonstrated that the samples clustered together by tissue of origin, not by individual animals (Figure 2). Gene expression patterns of the top 200 differentially expressed genes were hierarchically clustered using Euclidean distance, and the resulting dendrogram tree was cut into four groups (Figure 3). The genes, upregulated in the cells isolated from pericard and pleura (gene cluster 1), were enriched for genes associated with stem cell differentiation and organ formation, possibly indicating a higher level of multipotency. In contrast, trachea‐derived cells demonstrated a gene expression profile enriched for genes associated with the maintenance of cell polarity (gene cluster 4). In adipose‐derived progenitor cells, genes from several ontology terms, including neuron differentiation, cell morphogenesis regulation and embryonic organ morphogenesis, were significantly enriched, potentially indicating also these cells are substantially multipotent (gene cluster 3).

### 3.3 Multilineage differentiation

**FIGURE 4:**
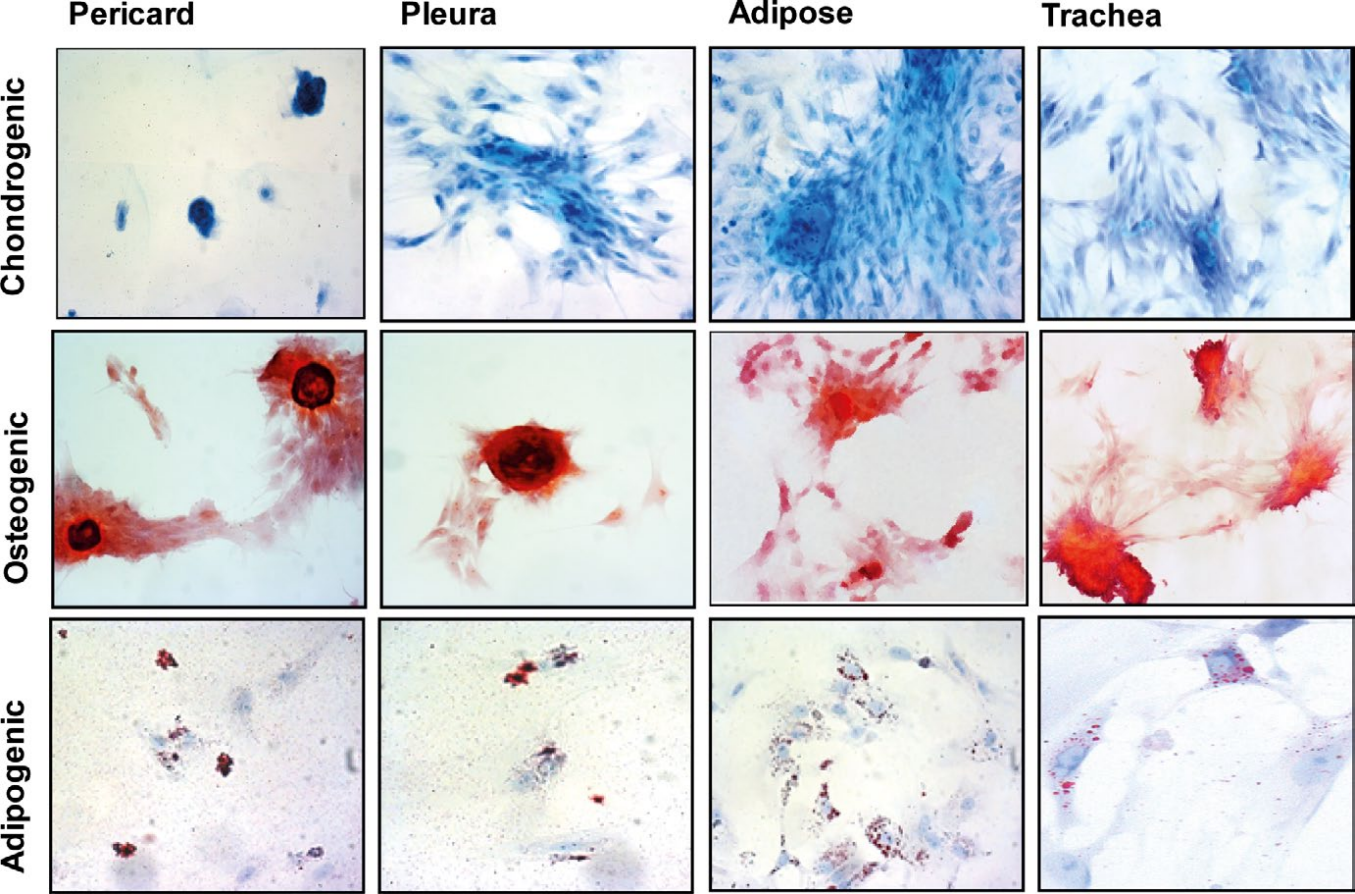
Isolated progenitor cells differentiate to mesenchymal lineages in vitro. Cells from pericard (A), pleura (B), adipose tissue (C) and trachea (D) all showed the capacity to adequately differentiate to chondrogenic (CH), osteogenic (OS) and adipogenic (AD) lineages. Differentiation did not occur in control groups. Chondrogenesis is indicated by glycosaminoglycan staining using Alcian blue. Osteogenesis was confirmed using Alizarin‐Red staining for calcium deposits. Accumulation of lipid vacuoles that stain with Oil Red O indicated adequate adipogenesis. Staining intensities were comparable. Magnification 400×. N = 3.

To confirm the multipotency of the cells, they were exposed to different chemical stimuli to induce chondrogenic, osteogenic and adipogenic cell differentiation. Regardless of the tissue of origin, cells could be induced along all three cell types: glycosaminoglycans deposition indicated successful chondrogenic differentiation; positive staining of calcium deposits by Alizarin Red confirmed adequate osteogenic differentiation; deposition of lipid droplets stained with Oil Red O confirmed adipogenic differentiation (Figure 4).

**FIGURE 5:**
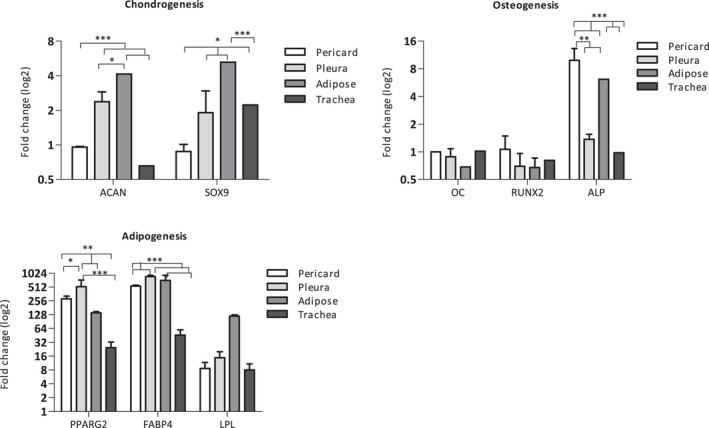
Normalized mRNA expression of chondrogenic, osteogenic and adipogenic associated genes after induction. Comparison of transcript levels of related genes in porcine isolated cells before and after differentiation for 14 days presented here in fold change (logarithmic scale). Significant differences are indicated with *p < 0.05. **p < 0.005. ***p < 0.001.

Expression of lineage‐specific molecules was examined by RT‐PCR (Figure 5). Pleura‐ and adipose‐derived cells, subjected to chondrogenic differentiation, showed upregulation of genes such as aggrecan (ACAN) (fold‐change PL 2.38; AD 4.14) and SOX9 (fold‐change PL 1.91; AD 5.27) as compared to undifferentiated control cultures, whereas ACAN and SOX9 expression levels did not change in pericardial‐derived cells. Tracheal cells showed a mixed pattern with lower ACAN levels, but higher SOX9 levels.

Cells subjected to osteogenic differentiation showed an increase of alkaline phosphatase (ALP) levels (fold‐change ALP PR 9.91; PL 1.38; AD 6.17; TR 0.97). In contrast, changes in osteocalcin (OC) and RUNX2 were limited and levels were equal or lower (fold‐change OC PR 1.00; PL 0.88; AD 0.68; TR 1.02) (fold change RUNX2 PR 1.07; PL 0.70; AD 0.68; TR 0.81).

Pericardial‐ and adipose‐derived cells expressed significantly higher activity of ALP after induction (*p *< 0.005 and *p *< 0.001 respectively), although differences in other tested genes were insignificant between the different sources.

Adipogenic differentiation stimuli resulted in substantial induction of adipose‐related genes: proliferator‐activated receptor‐γ2 (PPARγ2) (fold‐change PR 284.33; PL 521.50; AD 140.43; TR 24.61), fatty acid‐binding protein 4 (FABP4) (fold‐change PR 540.07; PL 863.95; AD 715.28; TR 45.64) and lipoprotein lipase (LPL) (fold‐change PR 8.70; PL 14.86; AD 119.23; TR 8.01). Pericard‐ and pleura‐derived cells had significantly improved PPARγ2 and FABP4 upregulation (*p *< 0.005 and *p *< 0.001 respectively), while adipose‐derived cells also expressed significant higher upregulation of FABP4 when compared to tracheal‐derived cells (*p *< 0.001).

Control groups did not show signs of differentiation. These findings indicated that isolated cell types contained subpopulations that have potential to differentiate in vitro towards mesenchymal phenotypes of bone, cartilage and/or adipose tissue, confirming stemcellness.^29^

### 3.4 Cell viability on scaffolds

**FIGURE 6:**
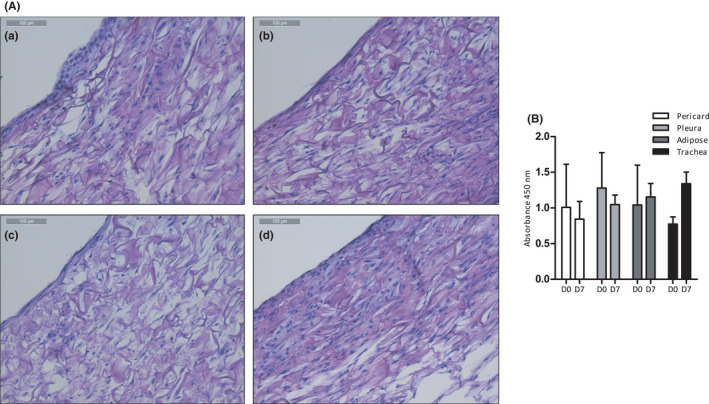
(A) Light microscopy images of haematoxylin and eosin stained cell seeded collagen scaffolds after 7 days. (a) Pericard‐derived cells. (b) Pleura‐derived cells. (c) Adipose‐derived cells. (d) Trachea‐derived cells. Magnification 200×. (B) Cell survival assessment after 7 days. Cell viability at day 0 (D0) and day 7 (D7) was analysed by WST assay. No significant differences between groups were observed. N = 3.

An important variable in tissue engineering is the ability of cells to survive and proliferate on a scaffold. Cell survival was assessed at day 0 and day 7 after seeding using the WST‐1 assay. In essence, cells were able to survive on the collagen scaffolds regardless of the cell type used. Cells grew into the scaffolds and proliferated. Slight differences in viability and proliferation were observed, but these were not significant. (Figure 6).

## 4 DISCUSSION

Creation of a sustainable tracheal substitute for tracheal repair after surgery remains a challenge. Most studies aimed at the development of tracheal substitutes, focus on new biomaterials with novel techniques.^30^ Although it is obvious that the designed matrix is of utmost importance, the importance of cell type and cell source seems to be underestimated. Evidence supports the benefits of graft cell seeding, particularly in the case of mesenchymal stem cells, but it remains unclear which specific cell types provide the most optimal regeneration.^31^

First, our data show that it is possible to isolate progenitor‐like cells from pericard, pleura and trachea as judged by expressed surface markers, gene expression and the possibility to differentiate along multiple lines depending on the chemical stimuli. The rather high variation in the relative expression of mesenchymal stem cell markers is most likely due to donor variation. To the best of our knowledge, this is the first report showing that progenitor cells can be isolated from these tissues. Mesenchymal stem cells or progenitor cells have been isolated from many sources, for example kidney, liver, amniotic fluid, synovium and umbilical cord, confirming the idea that these cells reside in the connective tissue of most organs.^32^ In general, these isolated cells were heterogeneous containing undifferentiated progenitors as well as lineage restricted precursors, and the potential to differentiate towards an osteogenic, adipogenic and chondrogenic lineage varied.^33^

Furthermore, the stem cell‐like character of the isolated cells is confirmed by their multilineage differentiation potential as confirmed by staining and marker expression. The morphological appearance of the isolated cells and glycosaminoglycan production, calcium deposition and lipid vacuoles accumulation was similar, regardless of the cell source. However, gene expression analysis after induction did reveal slight differences between the cells. Pericard‐derived cells failed to induce chondrocyte‐related genes, and induction of osteoblast‐related was limited. In contrast, pleura‐derived cells showed an inverse pattern: chondrocyte‐related genes were induced, and induction of osteoblast‐related genes was limited. Remarkably, all cells showed strong upregulation of adipose‐related genes.

To further delineate differences between the isolated cells and their possible preference to differentiate towards a particular cell type, we performed RNA‐sequencing and gene enrichment analysis. Isolated progenitor cells from pericard and pleura showed the most pronounced stem cell signature, a possible indication of their multipotency. Subsequently, the similarity of these cells may be a reflection of their shared embryonic origin. Based on the expression profiling, this may make them the superior choice for tracheal TE. Adipose‐derived progenitor cells showed a gene profile indicating multipotency as well, but the top 200 expressed genes were clearly dissimilar. Analysis revealed a segregation of the tissues based on the top differentially expressed genes, reflecting their different origins. Cells isolated from trachea showed a significant enrichment of genes associated with maintenance of cell polarity. This might be a reflection of far‐reaching cell commitment or a reflection of a very heterogeneous population, mainly consisting of fibroblasts with little relation to stem cells.

We attempted to isolate progenitor‐like cells from tracheal tissue, which likely resulted in a heterogeneous cell population in view of the isolation method: cells were isolated after outgrowth from small trachea segments, and under such conditions, contamination cannot be ruled out. However, phenotypic analysis showed the absence of cytokeratin‐positive epithelial cells, suggesting a reasonable homogeneous cell population. Not surprisingly, their gene expression signature differed substantially from the pleura‐, pericard‐ and adipose‐derived cells. Tracheal derived cells appeared to be more cell type‐committed. Yet, functional differentiation analysis showed that these cells still retained the capability to differentiate along three different lineages, suggesting that (a number of) these cells are still multipotent.

Because it is essential that cells can be seeded and proliferate on a tracheal scaffold, cell survival and proliferation on collagen scaffolds was evaluated. We observed survival and proliferation of all cells seeded on a collagen scaffold. Since collagen scaffolds do not have the required mechanical properties needed for sufficient strength, they might not be the optimal choice as a tracheal scaffold.^30^ More elaborate studies are needed to examine the potential of these cells on different scaffolds, such as often‐used decellularized trachea scaffolds, and to optimize cell seeding protocols for the most favourable promotion of regeneration in vivo.

One of the major drawbacks in the field of tracheal TE is ‘cell choice based on convenience’. In clinical practice, not all specialties have access to all cell sources. This, conceivably, leads to scientific manoeuvres based on the best available cell source. This phenomenon may potentially carry the roots for repetitive and perpetual failure of grafts on a long run. Our project is conducted from the department of cardio‐thoracic surgery. This provides a platform and opportunity to access cell sources based on fundamental hypotheses rather than accessibility threshold. Per patient, pleural and pericardial tissue can be obtained through a small thoracic or parasternal incision. Pleural tissue can be harvested through a small lateral incision under local anaesthesia, while pericardial tissue can be approached through VATS or a parasternal incision. This opens door to therapeutic options in a personalized fashion which potential long‐term sustainability based on cell origin and embryonic organ development.

Mesenchymal stem cells are a well‐accepted choice for cell seeding in tissue‐engineered organs due to their availability, capacity to expand in culture and multilineage differentiation capacity.^31^, ^34^, ^35^ Both bone marrow‐ and adipose‐derived stem cells are the most common used sources due to their easy accessibility, isolation potential and production of immunomodulatory factors.^18^, ^22^, ^23^ Unfortunately, when used in tracheal TE issues with mechanical failure, stenosis of grafts and anastomosis seem recurrent.^36^, ^37^, ^38^, ^39^, ^40^Thus, even though multipotent cells have been isolated from many different sources and showed the capacity for multilineage differentiation, their therapeutic potential might be different.^33^ In our case, differences between several progenitor cell sources mainly consisted of variation in gene signatures, whereas the functional analyses did not reveal outstanding differences.

We characterized new cell sources with a stem cell gene expression signature isolated from pericard and pleura, with cells able to differentiate along different lineages. Such cells may be valuable for tracheal TE approaches as they may be more easily committed to differentiate into the cells of interest (chondrocytes). It is acknowledged that the cell populations used may not have been homogenous and did not consist of pure stem cell populations. Under the here used conditions, observed differences are small. Current assays were unable to demonstrate significant differences within these populations, possibly overlooking a superior subpopulation that could lead to better functional outcome. A more homogeneous population of pleura and/or pericard‐derived cells may be superior in the context of, for instance the tracheal micromilieu.

Further studies are needed to better define the subpopulations present in the isolated cells. FACS sorting, based on stem cell markers such as CD117, may allow enrichment. Unfortunately, none of the isolated cell populations contained sufficient CD117+ cells for subsequent cell sorting (results not shown). Single‐cell RNA sequencing may lead to more insight in the various cell populations and define a marker usable for subsequent cell sorting to isolate purified stem cell populations. Since cell development and tissue remodelling is different in vivo with presence of the intricate microenvironment, in vivo research with these cell sources may provide a better long‐term outcome, when creating a sustainable tracheal construct that has proper mechanical strength, epithelization and vascularization potential.

## 5 STATEMENT OF SIGNIFICANCE

The ideal cell source in tracheal tissue engineering (TE) has yet to be determined. An alternative, possibly superior autologous cell source for cell seeding purposes was found in pleura‐ and pericard‐derived stem cells, based on their gene expression. These cells may be valuable for tracheal TE approaches as they may be more easily committed to differentiate into the cells of interest (chondrocytes), leading to better functional outcome of engineered constructs.

## CONFLICTS OF INTEREST

The authors indicate no potential conflicts of interest.

## AUTHOR CONTRIBUTION

**Rayna de Wit:** Conceptualization (equal); Data curation (lead); Formal analysis (lead); Investigation (lead); Writing‐original draft (lead). **Sailay Siddiqi:** Conceptualization (supporting); Formal analysis (supporting); Investigation (supporting); Supervision (supporting); Writing‐original draft (supporting). **Dorien Tiemessen:** Data curation (supporting); Formal analysis (supporting); Investigation (supporting); Writing‐original draft (supporting). **Rebecca Snabel:** Data curation (supporting); Formal analysis (supporting); Writing‐original draft (supporting). **Gert Jan Veenstra:** Data curation (supporting); Formal analysis (supporting); Supervision (supporting); Writing‐original draft (supporting). **Egbert Oosterwijk:** Conceptualization (supporting); Formal analysis (supporting); Investigation (supporting); Supervision (supporting); Writing‐original draft (supporting). **Ad Verhagen:** Conceptualization (supporting); Formal analysis (supporting); Investigation (supporting); Supervision (supporting); Writing‐original draft (supporting).

## Supporting information

Table S1‐S2

## Data Availability

The data that support the findings of this study are available from the corresponding author upon reasonable request.
