# Supplementary material for: Isolation of multipotent progenitor cells from pleura and pericardium for tracheal tissue engineering purposes
Source: J Cell Mol Med. 2021 Nov 1;25(23):10869–78. doi: 10.1111/jcmm.16916 (PMC8642678; doi:10.1111/jcmm.16916)
Supplement: Supplementary file 1 — Table S1‐S2 [file JCMM-25-10869-s001.docx]

**Supplementary table 1**

| *Gene* | *Primer sequences 5’🡪 3’* | *Product size, bp* | *Anneal temp. ‘C* | *Source^1^* |
| --- | --- | --- | --- | --- |
| *CD90* | F:GAACCCAGTATCTTTCAGGCATCTG  R: TGTCAGGCTGGTCACCTTCT | 199 | 61.76  60.43 | 927177037 |
| CD73 | F: CGCTTTCGGGAATGCAACAT  R: TTATTGTGCCATTGTTCCGCTC | 160 | 59.84  59.83 | 927094700 |
| CD105 | F: CTGGCCGTCTGCAGCTT  R: CCAGGGTTGACACCTCCTTT | 187 | 60.01  59.52 | 927099443 |
| CD117 | F: TACCAAGTGGCAAAGGGCAT  R: GGTAGCCGAGCGTTTCCTTT | 176 | 59.89  60.67 | 113205555 |
| SOX9 | F: AGGAAGTCGGTGAAGAACGG  R: GATGGCGTTGGGAGAGATGT | 75 | 59.68  59.82 | 47523169 |
| HPRT | F: GGGAGGCCATCACATCGTAG  R: CGCCCGTTGACTGGTCATTA | 167 | 59.97  60.39 | 1661109 |
| GAPDH | F: CCTCAACGACCACTTCGTCA  R: TGTGTTGGGGGATCGAGTTG | 199 | 59.97  59.96 | 329744641 |

^1^  Reference NCBI (GI)

**Supplementary table 2**

| *Gene* | *Primer sequences 5’🡪 3’* | *Product size, bp* | *Anneal temp. ‘C* | *Source^1^* |
| --- | --- | --- | --- | --- |
| RUNX2 | F: ATCACCTCAACCCTGGTTCT  R: GGGAGAACGCTGGCGATAC | 181 | 58.25  60.59 | 927157684 |
| ALP | F:ATTTCTCCAGACCCAGAAGCC  R: GGTCTCCCCTGCAGTTAGGA | 142 | 59.72  60.62 | 1036031607 |
| OC | F: TACCCAGATCCTCTGGAGCC  R: TGCCATAGAAGCGCCGATAG | 109 | 60.11  60.04 | 25245997 |
| PPARG2 | F:GTGTGAATGACAGCAAACCCC  R: GGTGTCAACCATGGTCACCT | 134 | 60.00  59.89 | 3646469 |
| FABP4 | F: ACGGCTTCTTTCTCACCTTGA  R: AGCCCACTCCCACTTCTTTC | 128 | 59.58  59.60 | 257467616 |
| LPL | F: AAGACTCGTGCTCAGATGCC  R: GGCAGGGTGAAAGGGATGTT | 152 | 60.11  60.25 | 55741568 |
| SOX9 | F: AGGAAGTCGGTGAAGAACGG  R: GATGGCGTTGGGAGAGATGT | 75 | 59.68  59.82 | 47523169 |
| ACAN | F: GTCCGCTCCTTGTCGCTTTC R: TGCATTTGCTTTGGCGTTTGA | 144 | 61.63  60.47 | 257467620 |

**^1^**Reference NCBI (GI)
